# Lung Cancer Staging at Diagnosis in the Veterans Health Administration: Is Rurality an Influencing Factor? A Cross-Sectional Study

**DOI:** 10.1111/jrh.12429

**Published:** 2020-05-30

**Authors:** Rolando Sanchez, Yunshu Zhou, Mary S. Vaughan Sarrazin, Peter J. Kaboli, Mary Charlton, Richard M. Hoffman

**Affiliations:** 1 Division of Pulmonary-Critical Care Medicine, Department of Internal Medicine, University of Iowa Carver College of Medicine, Iowa City, Iowa; 2 Veterans Rural Health Resource Center-Iowa City, Veterans Health Administration (VHA), Office of Rural Health, and the Center for Access and Delivery Research and Evaluation (CADRE) at the Iowa City VHA, Iowa City, Iowa; 3 Holden Comprehensive Cancer Center, University of Iowa, Iowa City, Iowa; 4 Institute for Clinical and Translational Science, University of Iowa, Iowa City, Iowa; 5 Department of Internal Medicine, Division of General Internal Medicine, University of Iowa Carver College of Medicine, Iowa City, Iowa; 6 Department of Epidemiology, University of Iowa College of Public Health, Iowa City, Iowa

**Keywords:** early-stage, late-stage, lung cancer, rural, veterans

## Abstract

**Purpose:**

To evaluate the association between rurality and lung cancer stage at diagnosis.

**Methods:**

We conducted a cross-sectional study using Veterans Health Administration (VHA) data to identify veterans newly diagnosed with lung cancer between October 1, 2011 and September 30, 2015. We defined rurality, based on place of residence, using Rural-Urban Commuting Area (RUCA) codes with the subcategories of urban, large rural, small rural, and isolated. We used multivariable logistic regression models to determine associations between rurality and stage at diagnosis, adjusting for sociodemographic and clinical characteristics. We also analyzed data using the RUCA code for patients’ assigned primary care sites and driving distances to primary care clinics and medical centers.

**Findings:**

We identified 4,220 veterans with small cell lung cancer (SCLC) and 25,978 with non-small cell lung cancer (NSCLC). Large rural residence (compared to urban) was associated with early-stage diagnosis of NSCLC (OR = 1.12; 95% CI: 1.00–1.24) and SCLC (OR = 1.73; 95% CI: 1.18–1.55). However, the finding was significant only in the southern and western regions of the country. White race, female sex, chronic lung disease, higher comorbidity, receiving primary care, being a former tobacco user, and more recent year of diagnosis were also associated with diagnosing early-stage NSCLC. Driving distance to medical centers was inversely associated with late-stage NSCLC diagnoses, particularly for large rural areas.

**Conclusions:**

We did not find clear associations between rurality and lung cancer stage at diagnosis. These findings highlight the complex relationship between rurality and lung cancer within VHA, suggesting access to care cannot be fully captured by current rurality codes.

Lung cancer is the second most frequently diagnosed cancer in the United States and by far the leading cause of cancer death.^1^ The overall 5-year relative survival among Americans diagnosed with lung cancer is only 18.6%.^1^ Survival is influenced by a number of factors, including stage at diagnosis,^1,2^ tumor histopathology,^3–5^ tumor biology,^6–8^ smoking and functional status,^9,10^ and treatment.^11–13^ Prognosis is dramatically worse for patients diagnosed with advanced stage cancers, small cell lung cancers, for patients with substantial comorbidities, and patients who do not undergo recommended treatment.

A mediating factor associated with survival appears to be geographic place of residence. Studies show increased lung cancer mortality among rural compared to urban populations.^14–16^ Some of this disparity may be related to geographic differences in receiving appropriate and timely treatment.^14^ This disparity could also be related to urban/rural differences in stage at diagnosis because earlier-stage cancers are more likely to be curable. Rural patients would be expected to have less access to health care services (including screening) and might present with more advanced stages at diagnosis. The studies assessing the influence of rurality in the stage at diagnosis in lung cancer, however, yielded conflicting results.^14,17–19^

The Veterans Health Administration (VHA) is an appropriate setting for evaluating geographic disparities in lung cancer stage at diagnosis. Lung cancer is the second most frequently diagnosed cancer among veterans,^20^ who also have higher rates of tobacco use than the general population.^21–24^ Furthermore, nearly one-third of the 8.2 million VHA enrollees live in rural areas.^25^ An integrated health care system, VHA provides full access to care for all enrolled veterans and captures comprehensive national demographic and clinical data in a common electronic health record. To our knowledge, there are no studies assessing the relationship between rural residence and lung (or other) cancer stage at diagnosis among the veteran population. Our overall objective was to investigate the associations between rural residency and stage at diagnosis for lung cancer in VHA. We hypothesized that rural veterans would have a less favorable lung cancer stage distribution at diagnosis than their urban counterparts.

## Methods

### Design: Cross-Sectional Study

#### Data Sources

We used national data extracted from the VHA Corporate Data Warehouse (CDW) to identify veterans newly diagnosed with lung cancer while enrolled in VHA between October 1, 2011, and September 30, 2015. The start date was to coincide with the publication of the National Lung Screening Trial, which demonstrated that low-dose CT (LDCT) screening reduced lung cancer mortality by 16% among heavy smokers ages 55 to 77, by increasing the detection of early-stage lung cancers.^26,27^ The end date was based on data availability and having a sufficient sample size. CDW data elements extracted from VHA’s integrated electronic health record and administrative files included patient demographics and residence, inpatient and outpatient visits, laboratory results, pharmacy records, and vital signs. The CDW oncology module contains elements from the VHA Cancer Registry, including cancer histology and diagnostic stage. Additional information on patient geography and primary site of care were obtained from the VHA Planning Systems Support Group (PSSG) geocoded veteran enrollment files and the Primary Care Management Module (PCMM), respectively.

#### Subjects

**Figure 1:**
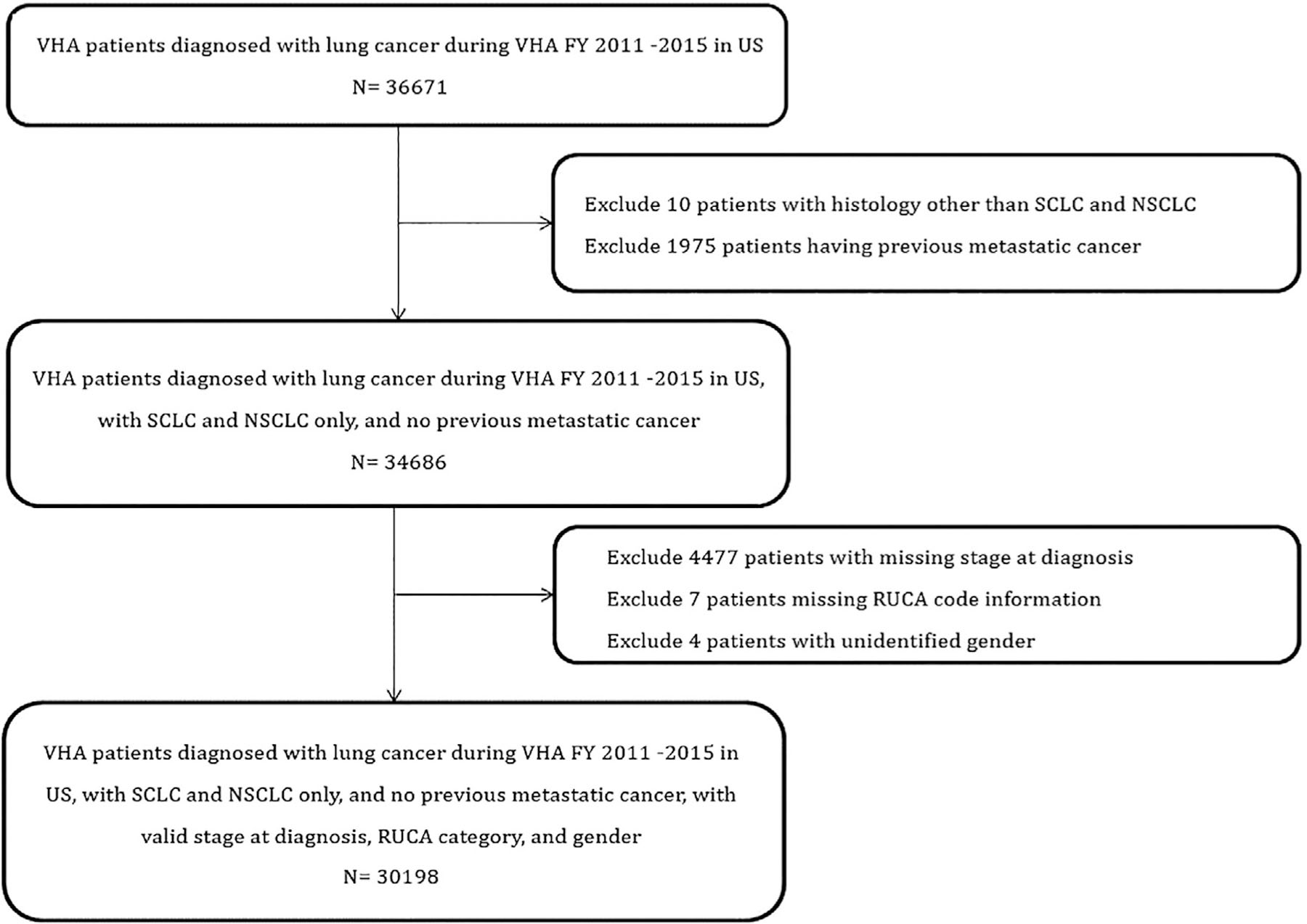
Cohort Selection and Exclusions.

We initially identified 36,671 veterans age ≥18 years who were diagnosed with lung cancer in a VHA facility within the 50 states of the United States (Puerto Rico or other US territories were excluded). Lung cancer was classified as small cell lung cancer (SCLC) and non-small cell lung cancer (NSCLC). Patients were excluded if the cancer histology was not small cell or NSCLC or missing (n = 10), had another recent diagnosis of other metastatic cancer prior to the lung cancer diagnosis (n=1,975), had missing diagnosis stage (n = 4,477), missing addree information (n = 7), or missing gender (n = 4). The final cohort of 30,198 VA patients with lung cancer included 4,220 with SCLC and 25,978 with NSCLC (Figure 1).

### Measures

#### Patient Characteristics

Patient characteristics included demographics, socioeconomic status, tobacco use, alcohol use, family history of cancer, comorbid conditions, and VHA health care utilization. Marital status was categorized as married, single/widowed/divorced, or unknown. Race and ethnicity were categorized as non-Hispanic white, black, other, and unknown. Tobacco use, alcohol use, and family history of cancer were identified in the CDW oncology module. Tobacco use was categorized as current or quit within 1 year, former (quit more than 1 year ago), former (quit date unknown), never used, and unknown. Alcohol use was categorized as current, past use, never use, and unknown. Previous chronic lung disease was identified using ICD-9-CM diagnosis codes (490–492.8, 493.00–493.92, 494.0–494.1, 495.0–505.x, 506.4) from inpatient and outpatient encounters during the 12 months prior to lung cancer diagnosis.^28^ We calculated the Charlson Comorbidity Index score using diagnoses from the 12 months before lung cancer diagnosis.^29^ We used an Index of Social Disadvantage to describe potential impacts of neighborhood social disadvantage on diagnosis.^30^ The index is a composite measure of 5 demographic characteristics collected in the American Community Survey: percentage of population living below the poverty line, (2) percentage of persons age 16 and older employed in professional and managerial occupations, (3) percentage of female-headed households, (4) percentage of males age 16 and older unemployed, and (5) median household income. The index is based on demographic characteristics summarized at the level of census tract.^30^ Higher scores indicate higher disadvantage. Finally, we identified the patient’s assigned VHA primary site of care from the PCMM and identified those with a primary care visit within 12 months before the diagnosis of lung cancer.

#### Geocoding

We defined residence using Rural-Urban Commuting Area (RUCA) codes, which classify US census tracts using 33 separate categories to represent population density, urbanization, and daily commuting.^25,31^ These 33 categories were condensed and grouped into 4 subcategories: urban, large rural, small rural, and isolated rural according to a recommended algorithm.^32^ RUCA codes were based on the patient’s address latitude and longitude coordinates, which were assigned to a specific census tract. For a small number of patients (10.6%), census tract was not identifiable and we used ZIP-code-based RUCA categories.^33^ Region of the country was defined using US Census Bureau regions (Northeast, South, West, and Midwest); see appendix (available online only).

#### Cancer Stage at Diagnosis

Stage data are reported using the American Joint Committee of Cancer criteria,^34,35^ with our primary endpoints being early and late-stage cancer at diagnosis. For patients with NSCLC, we defined early-stage as stage IA (ie, cancerous tumor is 3 cm across or smaller, does not affect the main bronchi, and has not spread to lymph nodes or other organs) because we were looking at differences in the rate of curable lung cancers between veterans living in urban and rural areas, and stage IA has the highest likelihood of cure with a 5-year survival up to 92%.^34^ We excluded patients (n = 185) with the nonspecific code of just stage I (ie, not stage IA or IB) from analysis of patients with NSCLC. For patients with SCLC, we considered patients diagnosed at either stage I, IA, or IB (lesions ≥3 cm without regional or distant metastasis) to have early (limited)-stage cancers. Late-stage NSCLC cancers were defined as stage IV (malignant pleural or pericardial effusion, cancer metastasis in a contralateral lung, or beyond the lungs into other areas of the body). All SCLC that were not early (limited) stage were considered to be late (extensive)-stage cancers.

### Statistical Analysis

We compared patients and tumor characteristics across the different RUCA subcategories, using analysis of variance (ANOVA) to compare continuous variables and used the chi-squared statistic to compare categorical variables. We then used multivariable logistic regression models to determine the association of rurality on stage at diagnosis. Separate models were generated to predict early-stage for NSCLC and SCLC and to predict late-stage for NSCLC. We did not model late-stage SCLC because this was just the inverse of the early-stage model. We modeled the subcategories of large rural, small rural, and isolated rural areas using urban as the reference category. We adjusted models for patient sociodemographic and clinical characteristics and region of the country. Models included random facility intercepts to control for facility-level variation in diagnostic staging. We tested for interactions between rurality and age, race/ethnicity, and region of the country.

We conducted several sensitivity analyses. First, we used the RUCA subcategory for the patient’s assigned primary care site; across the VHA, 65.8% of primary care sites are urban, 23.2% large rural, 9.5% small rural, and 1.5% isolated. Primary care clinic RUCA subcategories were assigned based on census tract, as identified from longitude and latitude coordinates of facilities available through the VHA site tracking (VAST) system.^36^ We did not look at the RUCA subcategory for the assigned tertiary care site because over 90% are in urban areas. We also used the driving distances from the patient’s residence to their assigned VHA primary care clinic and tertiary care center. We categorized driving distance as <40 miles, 40 to <120 miles, and ≥120 miles. The VHA Office of Planning Systems Support Group calculates driving distances to the VHA-assigned primary care and tertiary care centers for all enrolled veterans using actual longitude and latitude coordinates of their residences and the nearest VHA facilities. Travel distances are estimated using geospatial technologies that reflect available roads and average driving conditions. We also explored interactions between rurality and age, region, and race in the relative likelihood of late or early diagnosis. Finally, we generated multivariable models for stage at diagnosis stratified by region of the country. Models were fit using SAS Enterprise Guide v7.4 (SAS Institute Inc., Cary, NC). The Institutional Review Board at the Iowa City VHA approved this study.

## Results

Between October 1, 2011, and September 30, 2015, we identified 30,198 veterans with lung cancer, including 25,978 with NSCLC and 4,220 with SCLC (Figure 1). The most common histologic type of NSCLC was adenocarcinoma (42%) followed by squamous cell carcinoma (33%). We found no significant differences in histology across the RUCA subcategories. Table 1 shows baseline patient characteristics by RUCA subcategories. Notably, most lung cancer cases were diagnosed in veterans living in urban areas, with less than 10% of diagnoses in veterans residing in isolated or small rural areas. We found differences in sociodemographic characteristics across RUCA subcategories, but overall the cohorts largely comprised older, married white men with a history of tobacco use. Patients residing in large rural and small rural areas had the highest disadvantage index. About half of the patients had chronic lung disease and the average Charlson Comorbidity Index score was 1.8.

Table 2 shows the bivariable comparison of stage at diagnosis by rural residence. Patients living in large rural areas were most likely to present with early-stage lung cancer (NSCLC and SCLC). Only 21% of patients with NSCLC were diagnosed with stage IA. Less than a third of patients with SCLC were diagnosed at stage I. Nearly 40% of patients with NSCLC were diagnosed with stage IV cancer.

The multivariable odds for presenting with early-stage NSCLC (stage IA) and SCLC (stage I) are shown in Table 3. Among patients with SCLC, the RUCA subcategory of large rural was associated with an increased likelihood of being diagnosed at early-stage, compared to urban (OR = 1.73; 95% CI: 1.18–2.55). Having chronic lung disease was also associated with an early-stage diagnosis. Residing in a large rural area was associated with an increased likelihood of being diagnosed with early-stage NSCLC (OR = 1.12; 95% CI: 1.01–1.24, *P* = .028). Other factors associated with early-stage NSCLC included female gender, having chronic lung disease, a higher Charlson Comorbidity Index score, receiving VA primary care in the 12 months before diagnosis, being a former smoker, and diagnosis in a more recent year. Black race (compared to whites) and those age 80 and older (compared to those younger than 60) were significantly less likely to be diagnosed with early-stage NSCLC.

Table 4 shows factors associated with presenting with stage IV NSCLC. We found that veterans residing in large rural areas were less likely to present with advanced-stage NSCLC compared to those residing in urban areas (OR = 0.88; 95% CI: 0.80–0.95, *P* = .002). Other factors associated with lower likelihood of advanced-stage diagnosis include female sex, white race, age 65–79 (relative to age <60), having chronic lung disease, receiving VHA primary care in the 12 months before diagnosis, a higher Charlson Comorbidity Index score, being a former smoker (relative to current smokers), and diagnosis in a more recent year. Veterans who never smoked were significantly more likely to be diagnosed at an advanced stage.

We did not find any interactions between rurality and age or race in models of lung cancer stage at diagnosis (either early or late-stage), but we did find a significant interaction with region. In the analysis stratified by region, we found a protective effect for large rural areas that was present only in the South and Midwest regions. Specifically, patients living in Midwest large rural areas had significantly higher odds of early-stage diagnosis compared to urban counterparts for SCLC (OR = 2.30; 95% CI: 1.23–4.31) and NSCLC (OR = 1.27; 95% CI: 1.07–1.52). In contrast, patients in Western large rural areas were less likely to have early-stage NSCLC diagnosis than urban patients (OR = 0.71; 95% CI: 0.53–0.96). Finally, patients residing in Southern large rural areas were less likely to receive a late-stage NSCLC diagnosis than their urban counterparts (OR = 0.83; 95% CI: 0.72–0.94).

We also conducted sensitivity analyses using different measures of rurality than the RUCA subcategories for the patient’s residence. We found no associations between stage at diagnosis with either the primary care site RUCA subcategory or driving distance to the assigned VHA primary care clinic. In analyzing driving distance to the assigned VHA tertiary care center, we categorized distance as <40 miles (37% of veterans), 40 to <120 miles (37%), or ≥120 miles (26%). Compared to driving distances <40 miles, we found that longer driving distances were associated with lower likelihoods of a late-stage NSCLC diagnosis. For driving distances of 40–120 miles, the odds ratio (OR) was 0.92 (95% CI: 0.86–0.98), and the odds ratio was 0.82 (95% CI: 0.75–0.89) for a driving distance ≥120 miles. However, in stratified analysis by RUCA code, we found this effect was largely accounted for by the large rural population residing between 40 miles and 120 miles to the nearest tertiary care center having a greater likelihood of being diagnosed at early-stage NSCLC (OR = 1.20; 95% CI: 1.04–1.39). This population was also more likely to present with an early-stage SCLC (OR = 1.99; 95% CI: 1.15–3.45). In large rural areas, driving distances <40 miles were also associated with a decreased likelihood of being diagnosed with late-stage NSCLC (OR = 0.61; 95% CI: 0.41–0.91).

## Discussion

This large cohort study of 30,198 veterans diagnosed with lung cancer in 2011 through 2015 found that veterans living in large rural areas were more likely to be diagnosed with both early-stage SCLC and NSCLC compared to their urban counterparts. Veterans living in large rural areas were also less likely to present with advanced stage NSCLC.

We found no difference in the likelihood of being diagnosed with early-stage lung cancer between urban veterans and those living in small and isolated rural areas. These findings suggest that among veterans enrolled in the VHA, rurality, at least as coded by RUCA, may not be an important factor determining advanced stage of lung cancer at diagnosis, which is one of the most important predictors of clinical outcome.

The finding that veterans living in large rural areas were more likely to be diagnosed at an early stage with NSCLC and SCLC than those living in urban areas seems counterintuitive. However, a similar association has also been described in patients with lung and colon cancer in the non-VA population.^37,38^ Regional differences among rural and urban populations may account for some of these findings. For example, in some regions of the United States such as the Midwest, large rural populations could include large city suburbs, which may have better access to health care than the inner city urban population.^37^ Furthermore, the social disadvantage index, an important predictor of health-related outcomes among rural and urban populations, may also vary and even reverse according to region of the United States.^39^ Interestingly, our analysis by region showed that veterans living in large rural areas were more likely to be diagnosed with early-stage NSCLC and SCLC, and less likely to be diagnosed with late-stage NSCLC only in the Midwest and the South, respectively. The social disadvantage index also did not have a significant association with the stage at diagnosis in our study population. The sensitivity analyses suggest that differences observed in the rate of early-stage lung cancer diagnoses between urban and large rural veterans were not related to the location or the driving distance to the patient’s assigned primary care facility. However, increasing driving distance to the assigned VHA tertiary care center was associated with a more favorable lung cancer stage distribution at diagnosis, particularly for those living in rural areas and having a driving distance of 40–120 miles. This subset of patients would likely represent a suburban population.

Veterans living in rural areas are thought to experience several barriers to access health care, including provider and specialist shortages, hospital closings due to financial instability, geographic barriers, distance, and lack of transportation. Additionally, social factors like lower levels of housing, education, and employment may exacerbate these barriers.^25^ In this context, veterans living in rural areas would be expected to have lower rates of early-stage lung cancers. However, we did not observe any differences in the rate of early-stage lung cancer among veterans living in small rural and isolated rural areas compared to urban.

Studies in non-VA populations addressing the association between lung cancer stage at diagnosis and rurality have shown conflicting results.^14,40^ Shugarman and colleagues found no difference in the stage at diagnosis between lung cancer patients living in rural or urban areas among Medicare beneficiaries diagnosed between 1995 and 1999.^40^ Atkins and associates described similar findings when analyzing Surveillance, Epidemiology, and End Results Program data of lung cancer patients diagnosed between 2000 and 2006 in 18 states in the United States.^14^ A number of studies have also shown that rurality and longer driving times to diagnostic or treatment facilities are not clearly associated with advanced stage cancers, including for breast and colorectal.^41–44^ Henry and colleagues showed that geographic access was not associated with late-stage breast cancer when adjusted for poverty.^45^ However, Zahnd and associates and Silverstein and colleagues analyzed data from the North American Association of Central Cancer Registries (NAACCR) and the Savannah River Region Information System Cancer Registry, respectively. They found that patients living in rural areas and those with longer distance to the nearest hospital had slightly higher rates of advanced-stage lung cancers.^17,18^

Our study findings emphasize the complex relationship between rural residence and lung cancer outcomes. This association is determined at least partially by the classification system used to define rurality.^46,47^ While we used a 4-level classification, we might not have fully captured the diversity of geographic residences in United States. A specific-level classification of rurality could include populations with markedly different sociodemographic features depending on the US region of residence (South vs Midwest).^17^ Lung cancer stage at diagnosis may also have a marked heterogeneous geographic distribution within some large city populations.^37^ Factors like social disadvantage may also influence the relationship between health outcomes and urban-rural residence.^39^

The higher likelihood of being diagnosed with early-stage lung cancer in more recent years is probably related to the publication of the National Lung Screening Trial (NLST) results in 2011.^27^ The NLST demonstrated that screening high-risk individuals with LDCT compared to chest radiography increased the likelihood of detecting early-stage lung cancers and decreased lung-cancer-specific and overall mortality. Since then many professional societies,^48^ the US Preventive Services Task Force,^49^ and VHA^50^ have endorsed lung cancer screening with LDCT in high-risk populations. Currently, the VA is not mandating the implementation of lung cancer screening nationwide. However, primary care providers are highly encouraged to offer lung cancer screening to high-risk veteran patients. We would expect broader application of these guidelines in more recent years, with a subsequent increase in the rate of early-stage lung cancer.

The higher likelihood of early-stage lung cancer among NSCLC veteran patients with chronic lung disease, current alcohol use, receiving VHA primary care in the previous 12 months of diagnosis, and those with a higher Charlson Index score may be related to the higher frequency of health provider visits in this patient group. This increased interaction with health providers would translate into a higher chance of receiving radiological testing for their comorbidities, increasing the likelihood of early incidental lung cancer diagnoses.

Similar to other studies, we found that veterans with black race (compared to whites) were significantly less likely to be diagnosed with early-stage NSCLC.^51–54^ The association between black race and lower likelihood of early-stage lung cancer at diagnosis is poorly under-stood. Several factors including gaps in access and utilization of lung cancer screening programs,^55^ socioeconomic factors,^56^ and genetics and tumor biology^57^ may account for some of these differences. We did not find any significant interactions between race and rurality. We also found that those age 80 and older (compared to those younger than 60) were significantly less likely to be diagnosed with early-stage lung cancer. Since older patients usually have more frequent visits to primary care, one might expect this age group to be more likely to be diagnosed with early-stage cancers. However, professional society guidelines advise against lung cancer screening in patients of this age group, and if adopted, it would explain the lower likelihood of early-stage diagnosis among them.

Among patients with NSCLC, former smokers were less likely to be diagnosed early-stage compared to current smokers; never smokers were at higher risk of presenting with advanced stage than current smokers. A lower level of suspicion of cancer among never and former smokers could cause a delay in the diagnosis and explain the higher likelihood of advanced stage and lower likelihood of early-stage at diagnosis, respectively. Women were less likely to present with advanced stage NSCLC; the reason for this association is unclear, but it might be related to gender differences in tumor biology, endocrine factors, and smoking status.^24,58–60^

## Limitations

There are some limitations in our study. We were unable to account for veteran patients using non-VHA hospital services. It is unclear if the use of non-VHA hospital services affects the rate of detection of early-stage lung cancer. However, while about a third of VHA primary care clinics are located in large rural or small rural areas, over 90% of VHA tertiary care sites are urban. Utilization data suggest that very rural veterans appear to rely on VHA hospitals more than their urban counterparts.^61^ We could not account for individual-level variables like education and income that have been described to influence stage at diagnosis of lung cancer.^37^ However, we did at least include census-level data to assess social disadvantage. Because lung cancer mortality was not one of the outcomes in our study, we do not know whether the differences we observed for early-stage lung cancer diagnosis between large rural and urban veterans would translate into differences in lung cancer mortality. However, to our knowledge, this is the first study assessing the relationship between stage at diagnosis and place of residency among lung cancer patients in a veteran population.

## Conclusion

Veterans living in large rural areas of the United States were more likely to be diagnosed with early-stage NSCLC compared to their urban counterparts, though the association was significant only among those diagnosed in the South and Midwest. We found no difference in the likelihood of being diagnosed with early-stage lung cancer between veterans living in urban versus small and isolated rural areas. Our study findings suggest that the relationship between rurality and lung cancer within an integrated health care system is complex, and likely involves local health system, sociodemographic, and clinical factors that cannot be fully captured by using current rurality codes. Further studies are needed to understand the complex relationship between lung cancer outcomes and geographic variation with rural and urban populations in the United States and among veterans. Issues to address include better characterizing access to care by looking at patient-level factors, such as ability to obtain transportation, overall use of preventive services, comorbidity, sociodemographic data, and social networks. Health care delivery factors also play a role. In the case of lung cancer screening, many potentially eligible persons do not have ready access to screening centers, particularly high-risk persons in rural areas.^62^ Looking at rural-urban differences in accessing treatment, particularly curative, and survival differences would be other important outcomes to study.

## Supplementary Material

Supplementary Appendix

## Figures and Tables

**Table 1:** Patient Characteristics by Rural-Urban Commuting Area Code

	Urban	Large Rural	Small Rural	Isolated	Total	*P* values
Overall (%)	23,771 (78.7%)	3,276 (10.9%)	1,743 (5.8%)	1,408 (4.6%)	30,198	
Patient Characteristics						
Age (%)						<.0001
<60	3,223 (13.6%)	421 (12.9%)	193 (11.1%)	140 (9.9%)	3,977 (13.2%)	
60–64	5,475 (23%)	734 (22.4%)	389 (22.3%)	297 (21.1%)	6,895 (22.8%)	
65–69	6,039 (25.4%)	862 (26.3%)	465 (26.7%)	379 (26.9%)	7,745 (25.6%)	
70–74	3,436 (14.5%)	526 (16.1%)	272 (15.6%)	234 (16.6%)	4,468 (14.8%)	
75–79	2,649 (11.1%)	393 (12.0%)	199 (11.4%)	162 (11.5%)	3,403 (11.3%)	
≥80	2,949 (12.4%)	340 (10.4%)	225 (12.9%)	196 (13.9%)	3,710 (12.3%)	
Gender (%)						.39
Female	675 (2.8%)	80 (2.4%)	26 (1.6%)	26 (1.9%)	807 (2.7%)	
Male	23.096 (97.2%)	3,196 (97.6%)	1,717 (98.5%)	1,382 (98.2%)	29,391 (97.3%)	
Race (%)						<.0001
White	18,982 (79.9%)	2,891 (88.3%)	1,568 (90.0%)	1,305 (92.7%)	24,746 (82.0%)	
Black	4,216 (17.7%)	292 (8.9%)	128 (7.3%)	59 (4.2%)	4,695 (15.6%)	
Other	288 (1.2%)	39 (1.2%)	22 (1.3%)	31 (2.2%)	380 (1.3%)	
Unknown	285 (1.2%)	54 (1.7%)	25 (1.4%)	13 (0.9%)	377 (1.3%)	
Region (%)						<.0001
Northeast	3,326 (14.0%)	301 (9.2%)	103 (5.9%)	116 (8.2%)	3,846 (12.7%)	
South	9,950 (41.9%)	1,506 (46.0%)	793 (45.5%)	453 (32.2%)	12,702 (42.1%)	
Midwest	5,993 (25.2%)	1,020 (31.1%)	656(37.6%)	625 (44.4%)	8,294 (25.2%)	
West	4,502 (18.9%)	449 (13.7%)	191 (11.0%)	214 (15.2%)	5,356 (17.7%)	
Marital Status (%)						
Married	10,083 (42.4%)	1,627 (49.7%)	892 (51.2%)	745 (52.9%)	13,347 (44.2%)	<.0001
Single/Widowed/Divorced	13,651 (57.4%)	1,646 (50.2%)	851 (48.8%)	663 (47.1%)	16,811 (55.7%)	
Unknown	37 (0.2%)	3 (0.1%)	0 (0.0%)	0 (0%)	40 (0.1%)	
Tobacco History (%)						
Current/Quit ≤ 1 year	13,103 (55.1%)	1,817 (55.5%)	963 (55.3%)	758 (53.8%)	16,641 (55.1%)	.028
Former: Quit > 1 year	5,239 (22.0%)	757 (23.1%)	387 (22.2%)	368 (26.1%)	6,751 (22.4%)	
Former: Quit Date Unknown	2,787 (11.7%)	344 (10.5%)	214 (12.3%)	147 (10.4%)	3,492 (11.6%)	
Never Used	600 (2.5%)	84 (2.6%)	48 (2.8%)	34 (2.4%)	766 (2.5%)	
Unknown	2,042 (8.6%)	274 (8.6%)	131 (7.5%)	101 (7.2%)	2,548 (8.4%)	
Alcohol Use (%)						
Current Use	9,328 (39.2%)	1,136 (34.7%)	590 (33.9%)	508 (36.1%)	11,562 (38.3%)	<.0001
Past Use	5,310 (22.3%)	716 (21.9%)	393 (22.6%)	319 (22.7%)	6,738 (22.3%)	
Never	6,701 (28.2%)	1,063 (32.5%)	580 (33.3%)	434 (30.8%)	8,778 (29.1%)	
Unknown	2,432 (10.2%)	361 (11.0%)	180 (10.3%)	147 (10.4%)	3,120(10.3%)	
Chronic Lung Disease	11,534 (48.5%)	1,747 (53.3%)	916 (52.6%)	777 (55.2%)	14,974 (49.6%)	<.0001
Previous VA Primary Care Visit (Within Last 12 Months) (%)	21,525 (90.6%)	3,035 (92.6%)	1,597 (91.6%)	1,326 (94.2%)	27,483 (91.0%)	<.0001
Charlson Index (SD)	1.79 (1.4)	1.88 (1.4)	1.84 (1.4)	1.81 (1.4)		.31
Social Disadvantage Index (SD)	0.7 (3.5)	1.2 (2.6)	1.1 (2.1)	0.4 (1.9)	0.8 (3.4)	<.0001

**Table 2:** Bivariable Comparisons of Lung Cancer Stage at Diagnosis by Rural-Urban Commuting Area Code

	Urban (n = 23,771)	Large Rural (n = 3,276)	Small Rural (n = 1743)	Isolated (n = 1408)	Total (n = 30198)	*P* values
Small-cell lung cancer (%)	3,285 (13.8%)	444 (13.6%)	278 (16.0%)	213 (15.1%)	4,220 (14.0%)	
early-stage (I, IA, IB)	163 (5.0%)	38 (8.6%)	14 (5.0%)	11 (5.2%)	226 (5.4%)	.018
late-stage	3,122 (95.0%)	406 (91.4%)	264 (95.0%)	202 (94.8%)	3,994 (94.6%)	
Non-small cell lung cancer (%)	20,486 (86.2%)	2,832 (86.4%)	1,465 (84.0%)	1,195 (84.9%)	25,978 (86.0%)	
Stage I (all)*	5,898 (28.8%)	876 (30.9%)	410 (28.0%)	339 (28.4%)	7,523 (29.0%)	.089
Stage IA	4,218 (20.6%)	631 (22.3%)	278 (19.0%)	259 (21.7%)	5,386 (20.7%)	.001
Stage IB	1,537 (7.5%)	218 (7.7%)	123 (8.4%)	74 (6.2%)	1,952 (7.5%)	
Stage II	1,875 (9.2%)	265 (9.4%)	157 (10.7%)	123 (10.4%)	2,420 (9.3%)	
Stage III	4,609 (22.5%)	690 (24.4%)	355 (24.2%)	279 (243.4%)	5,933 (22.8%)	
Stage IV	8,104 (39.6%)	1,001 (35.4%)	543 (37.1%)	454 (38.0%)	10,102 (38.9%)	

* There were 185 patients with stage 1 diagnosis without further classification into 1A or 1.

**Table 3:** Factors Associated With Early-Stage Small Cell and Non-Small Cell Lung Cancer Diagnoses: Results from Multivariable Logistic Regression Models with Random Facility Effects.*

	Small Cell Lung Cancer (N = 4220)		Non-Small Cell Lung Cancer (N = 25,793)^†^	
		
Patient Characteristics	Odds ratio (95% CI)	*P* value	Odds ratio (95% CI)	*P* value
Rural Category (Reference: Urban)				
Isolated Rural	1.03 (0.54–1.97)	.92	1.08 (0.93–1.25)	.32
Small Rural	0.99 (0.55–1.76)	.97	0.91 (0.79–1.05)	.19
Large Rural	1.73 (1.18–2.55)	.005	1.12 (1.01–1.24)	.028
Female Sex (Reference: Male)	1.09 (0.49–2.43)	.83	1.85 (1.55–2.20)	<.001
Race (Reference: White)				
Black	1.12 (0.71–1.78)	.62	0.85 (0.77–0.93)	.001
Other	2.58 (1.05–6.35)	.04	0.91 (0.69–1.21)	.51
Unknown	0.38 (0.05–2.83)	.35	0.76 (0.56–1.03)	.07
Age Group (Reference: < 60)				
60–64	0.89 (0.52–1.51)	.66	0.98 (0.87–1.10)	.70
65–69	1.36 (0.82–2.26)	.24	1.07 (0.96–1.20)	.22
70–74	1.63 (0.94–2.80)	.08	1.08 (0.95–1.22)	.24
75–79	1.37 (0.74–2.52)	.32	1.06 (0.93–1.21)	.39
≥80	1.46 (0.80–2.69)	.22	0.77 (0.67–0.88)	<.001
Fiscal Year of Diagnosis (Reference: 2011)				
2012	1.16 (0.75–1.80)	.51	1.02 (0.92–1.13)	.68
2013	1.17 (0.74–1.84)	.51	1.08 (0.97–1.19)	.15
2014	1.20 (0.76–1.89)	.44	1.15 (1.03–1.27)	.009
2015	1.14 (0.69–1.88)	.60	1.24 (1.11–1.37)	<.001
Marital Status (Reference: Married)				
Single/Widowed/Divorced	1.02 (0.77–1.35)	.89	0.94 (0.88–1.00)	.04
Unknown	0.04 (0.00, ～)	.77	0.38 (0.11–1.27)	.12
Tobacco History (Reference: Current/Quit Within 1 year)				
Never Used	0.35 (0.05–2.61)	.30	1.20 (0.99–1.45)	.06
Former: Quit More Than 1 Year	1.10 (0.76–1.59)	.62	1.10 (1.01–1.19)	.02
Former: Quit Date Unknown	1.19 (0.74–1.90)	.48	1.13 (1.02–1.26)	.02
Unknown	0.92 (0.53–1.59)	.76	1.10 (0.96–1.25)	.18
Alcohol Use (Reference: never)				
Current Use	1.31 (0.91–1.88)	.15	1.09 (1.01–1.18)	.35
Past Use	1.22 (0.82–1.81)	.34	1.01 (0.93–1.11)	.80
Unknown	1.78 (1.10–2.86)	.02	1.01 (0.89–1.14)	.91
Chronic Lung disease (Reference: None)	1.67 (1.22–2.29)	<.001	1.25 (1.17–1.34)	<.001
Any Previous VA Primary Care (reference: none)	1.26 (0.70–2.28)	.45	2.19 (1.89–2.54)	<.001
Charlson Index	1.07 (0.97–1.19)	.17	1.12 (1.09–1.14)	<.001
Region (Reference: South)				
Northeast	0.90 (0.53–1.52)	.69	0.96 (0.79–1.17)	.68
Midwest	0.94 (0.64–1.39)	.77	0.94 (0.80–1.10)	.41
West	0.66 (0.40–1.09)	.10	1.01 (0.83–1.21)	.94
Disadvantage Score	0.99 (0.94–1.03)	.58	1.01 (1.00–1.02)	.15

* Small cell lung cancer: stage I; non-small cell lung cancer: stage IA.

† Excludes 185 patients with ambiguous stage I at diagnosis (ie, not stage IA or IB).

**Table 4:** Factors Associated With Stage IV Non-Small Cell Lung Cancer Diagnoses: Results From the Multivariable Logistic Regression Model With Random Facility Effects

	Non-Small Cell Lung Cancer (N = 25,978)	
Patient Characteristic	Odds Ratio, 95% CI	*P* value
Rural Category (Reference: Urban)		
Isolated Rural	0.96 (0.84–1.09)	.49
Small Rural	0.91 (0.81–1.02)	.11
Large Rural	0.88 (0.80–0.95)	.002
Female Sex (Reference = Male)	0.71 (0.60–0.84)	<.0001
Race (Reference: White)		
Black	1.22 (1.13–1.32)	<.0001
Other	1.15 (0.92–1.45)	.22
Unknown	1.34 (1.07–1.68)	.01
Age Group (Reference: <60)		
60–64	0.96 (0.88–1.05)	.39
65–69	0.86 (0.79–0.94)	.001
70–74	0.87 (0.79–0.96)	.007
75–79	0.88 (0.80–0.99)	.026
≥ 80	1.03 (0.93–1.15)	.56
Fiscal Diagnosis Year (Reference: 2011)		
2012	0.92 (0.85–1.00)	.056
2013	0.93 (0.86–1.01)	.082
2014	0.91 (0.84–0.99)	.032
2015	0.78 (0.72–0.86)	<.001
Marital Status (Reference: Married)		
Single/Widowed/Divorced	1.04 (0.98–1.09)	.19
Unknown	2.10 (1.04–4.24)	.038
Tobacco History (Reference: Current/Quit Within 1 Year)		
Former: Quit More Than 1 Year	0.93 (0.87–0.99)	.03
Former: Quit Date Unknown	0.97 (0.88–1.06)	.51
Never Used	1.28 (1.09–1.49)	.002
Unknown	0.97 (0.87–1.08)	.59
Alcohol Use (Reference: Never)		
Current Use	0.97 (0.87–1.07)	.39
Past Use	1.00 (0.93–1.07)	.94
Unknown	1.02 (0.88–1.08)	.65
Chronic Lung Disease (reference = No)	0.75 (0.71–0.79)	<.001
Previous VA Primary Care (Reference: No)	0.64 (0.58–0.70)	<.001
Charlson Index	0.91 (0.89–0.93)	<.001
Region (Reference: South)		
Northeast	1.14 (0.98–1.33)	.08
Midwest	1.12 (0.99–1.26)	.07
West	1.09 (0.95–1.25)	.23
Disadvantage Score	0.99 (0.98–1.00)	.005
