# Supplementary material for: Lung Cancer Staging at Diagnosis in the Veterans Health Administration: Is Rurality an Influencing Factor? A Cross-Sectional Study
Source: J Rural Health. Author manuscript; Available in PMC 2022 Feb 24. (PMC8867495; doi:10.1111/jrh.12429)
Supplement: Supplementary Appendix [file NIHMS1773254-supplement-Supplementary_Appendix.docx]

**Appendix**

**RUCA Code Definitions - WWAMI Rural Health Research Center**

1 Metropolitan area core: primary flow within an Urbanized Area (UA)

1.0 No additional code

1.1 Secondary flow 30% to 50% to a larger UA

2 Metropolitan area high commuting: primary flow 30% or more to a UA

2.0 No additional code

2.1 Secondary flow 30% to 50% to a larger UA

3 Metropolitan area low commuting: primary flow 10% to 30% to a UA

3.0 No additional code

4 Micropolitan* area core: primary flow within an Urban Cluster (UC) of 10,000 through 49,999 (large UC)

4.0 No additional code

4.1 Secondary flow 30% to 50% to a UA

5 Micropolitan* high commuting: primary flow 30% or more to a large UC

5.0 No additional code

5.1 Secondary flow 30% to 50% to a UA

6 Micropolitan* low commuting: primary flow 10% to 30% to a large UC

6.0 No additional code

7 Small town core: primary flow within an Urban Cluster of 2,500 through 9,999 (small UC)

7.0 No additional code

7.1 Secondary flow 30% to 50% to a UA

7.2 Secondary flow 30% to 50% to a large UC

8 Small town high commuting: primary flow 30% or more to a small UC

8.0 No additional code

8.1 Secondary flow 30% to 50% to a UA

8.2 Secondary flow 30% to 50% to a large UC

9 Small town low commuting: primary flow 10% through 29% to a small UC

9.0 No additional code

10 Rural areas: primary flow to a tract outside a UA or UC (including self)

10.0 No additional code

10.1 Secondary flow 30% to 50% to a UA

10.2 Secondary flow 30% to 50% to a large UC

10.3 Secondary flow 30% to 50% to a small UC

**UA=Urbanized Area**

**UC=Urban Cluster**

*The county-based term “micropolitan” was introduced by the Census Bureau per the 2000 Census. These Urban Clusters of from 10,000-49,999 population have historically been counted as Non-Metropolitan (OMB definition) and their definition has not substantially changed. The term is used in this table for consistency. Alternatively the term “[Large Rural](https://depts.washington.edu/uwruca/RUCACodeDes2.pdf)” or “Large Rural City/Town” could be substituted for “micropolitan” (the “Large Rural” link provides a pdf that includes the substitution). If the Census Bureau had taken a less urban centric view, they might have named the “micropolitan” category something more rural oriented like “macro-rural”. In addition, on the alternate table “small town” is labeled “small rural town” and “rural areas” is labeled “isolated small rural areas”.

**Classification of RUCA**

Urban (RUCA codes 1.0, 1.1, 2.0. 2.1, 3.0, 4.1, 5.1, 8.1, 10.1)

Large Town (RUCA Codes 4.0, 4.2, 5.0, 5.2, 6.0, 6.1)

Small Town (RUCA Codes 7.0, 7.2, 8.0, 8.2, 9.0, 9.1, 9.2)

Rural (RUCA code 10.0, 10.1, 10.2, 10.3)
